# Prediction of mucin-type O-glycosylation sites in mammalian proteins using the composition of *k*-spaced amino acid pairs

**DOI:** 10.1186/1471-2105-9-101

**Published:** 2008-02-18

**Authors:** Yong-Zi Chen, Yu-Rong Tang, Zhi-Ya Sheng, Ziding Zhang

**Affiliations:** 1 Bioinformatics Center, College of Biological Sciences, China Agricultural University, 100094 Beijing, China; 2 National Institute of Biological Sciences, No. 7 Science Park Road, Beijing 102206, China

## Abstract

**Background:**

As one of the most common protein post-translational modifications, glycosylation is involved in a variety of important biological processes. Computational identification of glycosylation sites in protein sequences becomes increasingly important in the post-genomic era. A new encoding scheme was employed to improve the prediction of mucin-type O-glycosylation sites in mammalian proteins.

**Results:**

A new protein bioinformatics tool, CKSAAP_OGlySite, was developed to predict mucin-type O-glycosylation serine/threonine (S/T) sites in mammalian proteins. Using the composition of *k*-spaced amino acid pairs (CKSAAP) based encoding scheme, the proposed method was trained and tested in a new and stringent O-glycosylation dataset with the assistance of Support Vector Machine (SVM). When the ratio of O-glycosylation to non-glycosylation sites in training datasets was set as 1:1, 10-fold cross-validation tests showed that the proposed method yielded a high accuracy of 83.1% and 81.4% in predicting O-glycosylated S and T sites, respectively. Based on the same datasets, CKSAAP_OGlySite resulted in a higher accuracy than the conventional binary encoding based method (about +5.0%). When trained and tested in 1:5 datasets, the CKSAAP encoding showed a more significant improvement than the binary encoding. We also merged the training datasets of S and T sites and integrated the prediction of S and T sites into one single predictor (i.e. S+T predictor). Either in 1:1 or 1:5 datasets, the performance of this S+T predictor was always slightly better than those predictors where S and T sites were independently predicted, suggesting that the molecular recognition of O-glycosylated S/T sites seems to be similar and the increase of the S+T predictor's accuracy may be a result of expanded training datasets. Moreover, CKSAAP_OGlySite was also shown to have better performance when benchmarked against two existing predictors.

**Conclusion:**

Because of CKSAAP encoding's ability of reflecting characteristics of the sequences surrounding mucin-type O-glycosylation sites, CKSAAP_ OGlySite has been proved more powerful than the conventional binary encoding based method. This suggests that it can be used as a competitive mucin-type O-glycosylation site predictor to the biological community. CKSAAP_OGlySite is now available at .

## Background

Representing one of the most common but complicated protein post-translational modifications (PTMs), protein glycosylation is abundant in many cell surface and secreted eukaryotic proteins [1-3]. Glycosylation is involved in a variety of important biological processes including protein stability, solubility, secretion of signal, regulation of interactions, extracellular recognition, etc [2]. Glycosylation is also strongly associated with marketed therapeutic proteins, since more than one-third of approved biopharmaceuticals are glycoproteins [3].

The detection of glycosylation sites in a query protein is very helpful to understand its biological function. Compared with the huge number of known protein sequences obtained from genomic and proteomic studies, the experimentally identified glycosylation sites are still limited. Proteomics analysis of glycoproteins by mass spectrometry (MS) is very promising to speed up the experimental identification of glycosylation sites [2]. Meanwhile, computational detection of glycosylation sites is also playing an increasingly important role [4,5].

N-linked and O-linked are two major types of glycosylation. N-linked glycosylation (N-glycosylation) is characterized by the β-glycosylamine linkage of N-acetylglucosamine (GlcNac) to asparagine (Asn) [1]. It has been well established that the consensus sequence motif Asn-X-Ser/Thr is essential in N-glycosylation [6]. The most abundant form of O-linked glycosylation (O-glycosylation), called "mucin-type", is characterized by α-N-acetylgalactosamine (GalNac) attached to the hydroxyl group of serine/threonine (Ser/Thr) side chains [7,8]. Therefore, S and T (*i.e*. the one-letter abbreviations of serine and threonine) are regarded as mucin-type O-glycosylation sites. Mucin-type O-glycosylation is commonly found in many secreted and membrane-bound mucins in mammal, although it also exists in other higher eukaryotes [8,9]. As the main component of mucus, a gel playing crucial role in defending epithelial surface against pathogens and environmental injury, mucins are in charge of organizing the framework and conferring the rheological property of mucus. Beyond the above properties exhibited by mucins, mucin-type O-glycosylation is also known to modulate various protein functions in vivo [7]. For instance, mucin-like glycans can serve as receptor-binding ligands during an inflammatory response [10]. Unlike N-glycosylation, the consensus motif has not been identified in the sequence context of O-glycosylation sites. Thus, computational prediction of mucin-type O-glycosylation sites in mammalian proteins is challenging and has received considerable attention. Prediction of O-glycosylation sites could offer valuable information for characterizing a new protein's functional and structural properties, like explaining mass spectrometry results as well as improving protein structure prediction [8]. Considering the roles of mucin-type O-linked glycoproteins involved in different diseases, computational identification of O-glycosylation sites can also be helpful in drug design [7]. In the current study, we focus on developing a new algorithm to detect mucin-type O-glycosylation sites in mammalian proteins.

A series of important prediction methods for mucin-type O-glycosylation sites have been elegantly developed. In 1993, Elhammer *et al*. used a matrix statistics method to initiate the prediction of O-glycosylation sites [11]. Subsequently, a vector projection method was developed [12,13]. Furthermore, a few state-of-the-art machine learning methods such as Neural Network (NN) and Support Vector Machine (SVM) were also heavily employed to perform the prediction [8,14-18]. Some well-maintained O-glycosylation site prediction web-servers, such as NetOGlyc 3.1 [8], are also publicly available. Even so, the prediction accuracy of these methods is generally not high enough. Some methods revealed less convincing performance when benchmarked with independent experimental studies [19-21]. Therefore, development of more accurate O-glycosylation site predictor is required.

The input feature vector (*i.e*. encoding scheme) is very important in obtaining a machine learning algorithm based predictor. Generally the input for an O-glycosylation site predictor is presented by a 2*n*+1 residue long sequence with S or T in the center (*i.e*. the window size is equal to 2*n+*1). The common position-specific features such as the standard binary encoding have been widely used as input features [8,15,18]. Some predicted structural properties like the solvent accessibility and secondary structure of a glycosylation site's sequence context were also used as input features [8,16]. Another possible useful encoding is the evolutionary information in the form of multiple sequence alignment profiles generated by PSI-BLAST program [22], which has also been integrated into the NetOGlyc 3.1 [8]. Parallel to the method development of O-glycosylation site prediction, the sequence and structural characters of O-glycosylation sites were also investigated [14,23,24]. These analyses are very helpful in guiding the selection of new encoding scheme to predict O-glycosylation sites.

In the present study, the prediction of O-glycosylation sites was improved by seeking new encoding schemes. After evaluating different encoding schemes, it was found that the composition of *k*-spaced amino acid pairs (CKSAAP) is suitable for representing an O-glycosylation site's sequence context. The CKSAAP reflects the short-range interactions of amino acids within a sequence or sequence fragment, which has been successfully employed for the prediction of protein flexible/rigid regions [25] and protein crystallization [26]. When *k *= 0, the CKSAAP reduces to the dipeptide composition, which has been applied in diverse prediction topics in the field of protein bioinformatics [27-29]. With the assistance of SVM, a predictor named CKSAAP_OGlySite has been set up to detect mucin-type O-glycosylation sites in mammalian proteins. The proposed encoding scheme resulted in a higher accuracy than the conventional binary encoding. The details about this proposed predictor are reported and the overall performance is benchmarked against two existing predictors.

## Results and Discussion

### Prediction Performance

To develop a new O-glycosylation site predictor, mammalian proteins containing experimentally verified mucin-type O-glycosylation sites were collected from the Swiss-Prot database [30]. The verified O-glycosylated S and T sites were compiled into positive sites (i.e. positive datasets), while those S and T residues in these proteins with no annotation related to O-glycosylation site were selected as non-glycosylation sites (i.e. negative datasets). Represented by a sequence fragment with central S or T residue, each site was further parameterized by using the CKSAAP encoding scheme. CKSAAP_OGlySite predictor was then constructed with the assistance of SVM algorithm. CKSAAP_OGlySite was trained in datasets with two different ratios of O-glycosylation and non-glycosylation sites (i.e. 1:1 and 1:5) and tested by using a 10-fold cross-validation. More details about the compilation of datasets, CKSAAP encoding and SVM algorithm are outlined in the Methods section. Four measurements, i.e. Accuracy (*Ac*), Sensitivity (*Sn*), Specificity (*Sp*) and Matthew correlation coefficient (*MCC*), were jointly used to assess the performance of the proposed O-glycosylation site predictor (*cf*. Table 1). When balanced datasets (i.e. the ratio of O-glycosylation to non-glycosylation site was 1:1) were used, the CKSAAP encoding with and without feature selection were considered due to its high dimensionality, thus different SVM models were obtained. The adopted feature selection methods were correlation coefficient (CC-) and information entropy (IE-) based methods. Generally, the kernel of radial basis function (RBF) resulted in an optimal accuracy, although different parameters were optimized in different SVM models. In these models, the window size was preliminarily optimized to be set as 19 (*i.e*. 2*n*+1 = 19) and *k*_*max *_was also optimally set as 4 (*i.e*. the *k*-spaced amino acid pairs were considered for *k *= 0, 1, 2, 3 and 4). The CKSAAP encoding with a CC-based feature selection resulted in the highest accuracy in predicting O-glycosylation sites. The overall prediction accuracy (*Ac*) reached 83.1% for S (*Sn *= 80.7%, *Sp *= 85.6%, *MCC *= 0.671) and 81.4% for T (*Sn *= 80.3%, *Sp *= 82.5%, *MCC *= 0.632), which is almost equal to the IE-based feature selection and slightly better than the CKSAAP encoding without feature selection (*cf*. Table 1).

**Table 1:** Prediction accuracy of O-glycosylation sites based on different encoding schemes^a^

Site	Encoding scheme	Feature selection	*Sn *(%)	*Sp *(%)	*Ac *(%)	*MCC*
S	Binary^b^	No selection	74.2 ± 1.7	81.9 ± 3.0	78.0 ± 1.9	0.567 ± 0.039
	Binary^c^	No selection	76.5 ± 3.5	74.6 ± 3.6	75.6 ± 3.1	0.523 ± 0.060
	CKSAAP	No selection	77.9 ± 1.7	86.5 ± 3.0	82.2 ± 1.8	0.655 ± 0.037
	CKSAAP^c^	No selection	79.0 ± 5.2	83.0 ± 2.4	81.0 ± 2.6	0.628 ± 0.050
	CKSAAP	CC	80.7 ± 3.3	85.6 ± 3.9	**83.1 ± 2.8**	**0.671 ± 0.055**
	CKSAAP	IE	82.1 ± 2.3	83.9 ± 3.8	83.0 ± 2.4	0.665 ± 0.048

T	Binary^b^	No selection	74.8 ± 4.1	78.3 ± 1.7	76.6 ± 2.3	0.536 ± 0.045
	Binary^c^	No selection	77.8 ± 3.4	76.6 ± 3.2	77.2 ± 2.4	0.548 ± 0.048
	CKSAAP	No selection	80.4 ± 2.2	82.3 ± 2.9	81.3 ± 2.3	0.631 ± 0.045
	CKSAAP^c^	No selection	80.3 ± 1.9	85.7 ± 1.9	83.0 ± 1.8	0.666 ± 0.038
	CKSAAP	CC	80.3 ± 1.8	82.5 ± 2.3	**81.4 ± 1.3**	**0.632 ± 0.026**
	CKSAAP	IE	80.8 ± 1.5	81.9 ± 3.1	81.3 ± 2.2	0.631 ± 0.045

^a ^The SVM based prediction algorithm with the RBF kernel function. The CC-based feature selection resulted in the highest accuracy, and the corresponding values of *Ac *and *MCC *were represented in bold types. The corresponding measurement was represented as the average value ± standard deviation. ^b ^In this encoding scheme, the window size was optimally set as 41. ^c^The method was trained and tested on new negative site data sets where <40% identity was not required between positive and negative sites.

The result based merely on balanced datasets is not sufficient to evaluate the performance of an encoding scheme because of the fact that there are much more non-glycosylation sites than O-glycosylation sites in mammalian proteins. To have a reliable evaluation of the CKSAAP encoding, the proposed predictor was also carried out using 1:5 datasets with the same window size and *k*_*max *_as used in balanced datasets. Considering the minor contribution resulted from the dimensional reduction in dealing with balanced datasets, feature selection was not carried out in this case. When the numbers of positive and negative data are different, *MCC *should be more suitable for assessing the overall prediction accuracy. The value of *MCC *ranges from -1 to 1, and higher *MCC *stands for better prediction performance. As shown in Table 2, the *MCC *value reached 0.575 for S (*Sn *= 56.7%, *Sp *= 95.6%, *Ac *= 89.1%) and 0.608 for T (*Sn *= 68.8%, *Sp *= 92.9%, *Ac *= 88.9%). Methods based on different ratios of positive to negative sites were reported in the literature [8,18]. The performance of CKSAAP_OGlysite in this study is based merely on datasets with two different ratios. To construct a better predictor for practical use, the ratio may be further optimized by evaluating the algorithm in all the mammalian proteins with verified O-glycosylation sites. However, the number of experimentally determined O-glycosylation sites is still quite limited and the real proportion of O-glycosylation to non-glycosylation sites in mammalian proteins is still unclear. Therefore, how to select the optimal ratio of positive/negative sites in training a prediction model remains an open question. Since the ratio of positive/negative sites in NetOGlyc3.1 is close to 1:5, in this paper a ratio of 1:5 dataset was selected to allow a fair comparison between CKSAAP_OGlySite and NetOGlyc3.1.

**Table 2:** Comparison of CKSAAP_OGlySite with NetOGlyc 3.1

Site	Method	*Sn *(%)	*Sp *(%)	*Ac *(%)	*MCC*
S	Binary^a,b^	49.7 ± 4.8	88.0 ± 0.8	81.7 ± 1.4	0.364 ± 0.054
	CKSAAP_OGlySite^a,b^	56.7 ± 3.2	95.6 ± 0.4	89.1 ± 0.8	0.575 ± 0.040
	NetOGlyc 3.1^b^	54.9 ± 0.3	91.6 ± 0.7	85.6 ± 0.5	0.473 ± 0.011

T	Binary^a,b^	60.8 ± 0.8	85.4 ± 1.3	81.3 ± 1.2	0.416 ± 0.026
	CKSAAP_OGlySite^a,b^	68.8 ± 1.7	92.9 ± 0.3	88.9 ± 0.2	0.608 ± 0.009
	NetOGlyc 3.1^b^	76.9 ± 0.0	86.1 ± 0.6	84.6 ± 0.5	0.549 ± 0.009

^a^The method was trained and tested in datasets with a 1:5 ratio of O-glycosylation sites to non-glycosylation sites. ^b ^The corresponding measurement was represented as the average value ± standard deviation.

The negative dataset may contain numerous un-annotated positive sites, which is one of the major limitations of the machine learning based O-glycosylation site predictors. To remove these "potential" O-glycosylation sites within the data sets of negative sites, those with >40% identity with any positive site were discarded. The definition of the identity between two sites is detailed in the section of Datasets. Based on this strategy, some "true" negative sites with relatively high sequence identity with any positive site were filtered. Thus, it seems that only the "easy" negative sites remain in the training datasets, then one may argue that such a filtration may "artificially" result in a higher performance. To clarify this point, we performed another computational experiment by selecting negative sites without the filtration of 40% identity, and then the proposed prediction method was re-trained and assessed. As shown in Table 1, the predictor based on the new negative datasets only cause a minor difference of accuracy (-1.2% in predicting S sites and +1.7% in predicting T sites). Therefore, the filtration of 40% identity did not result in an overestimated accuracy.

### Top ranked amino acid pairs

The limited improvement resulted from the dimensional reduction (+0.9% for S sites and +0.1% for T sites) is probably because SVM has a good tolerance to high dimensional data. In other words, SVM is not sensitive to the so-called "the curse of dimensionality". The dimensionality reduction was able to allow us to catch a glimpse at those "important" amino acid pairs remained after the CC- or IE-based feature selection. To guarantee the 10-fold cross-validation is a real cross-validation, the rule of dimensionality reduction is strictly limited to be inferred from the training data set. In the 10-fold cross-validation, therefore, the final dimensionality is different in different SVM models. With a cut-off value of 0.1 in the CC-based feature selection, the final dimensionality are in the range from 490–510 for S and 300–330 for T. Regarding the IE-based feature selection, the cut-off of *IG *was set as 0.1 and the final dimensionality is around 640–660 for S, 730–750 for T. Based on all the cross-validation tests, the top 20 *k*-spaced amino acid pairs after the dimensionality reduction were listed in Table 3. Although two different feature selection methods resulted in two different subsets of the selected features, they share some common amino acid pairs, implying a good consistency between these two methods. In the top 20 features of O-glycosylated S sites, the CC- and IE-based methods resulted in 15 consistent amino acid pairs, whereas the number of consistent amino acid pairs is 12 in analyzing O-glycosylated T sites.

**Table 3:** The top 20 features selected by correlation coefficient (CC-) and information entropy (IE-) based methods

Site	S		T	
Top 20 features	CC	IE	CC	IE

1	**ST**	**PXXS**	**TXXP**^a,b^	**TXXP**
2	**SXXP**	**PXP**	**PT**	**PT**
3	**PXXXXS**	**PS**	AXXXP	**TT**
4	**PXXS**	SXS	**PXT**	TXXXT
5	**TXXP**	**SXXP**	**PP**	TXXXXT
6	**PXP**	**ST**	**TP**	TXT
7	**PXXXXP**	**SXXXP**	AXP	**PXT**
8	**SXXXP**	**PXXXP**	**PXXXXP**	**TP**
9	**SXP**	SXXXS	**TXXXXP**	**PP**
10	TXXXP	**PXXXXP**	TXA	**PXP**
11	**PS**	**PXXXXS**	**PXXXP**	TS
12	SXXXT	SXXXXP	SXXXP	**TXXXXP**
13	TP	**TS**	**SXXT**	**PXXXXP**
14	**TXXXXS**	SS	**TT**	**SXXT**
15	**TS**	SP	PXXA	**PXXXP**
16	TXXA	**SXP**	SA	SXXXXT
17	**PXT**	**PXT**	PXA	TXXS
18	**PXXXP**	**TXXXXS**	**TXXXP**	ST
19	PP	**TXXP**	**PXP**	**TXXXP**
20	**PXXP**	**PXXP**	AXXXXT	PXXXT

^a ^TXXP represents a 2-spaced amino acid pair of TP, where X stands for any amino acid. The same representation was applied to other *k*-spaced amino acid pairs. ^b ^The *k*-spaced amino acid pairs in bold type mean they are consistently ranked as the top 20 features by both feature selection methods.

**Figure 1:**
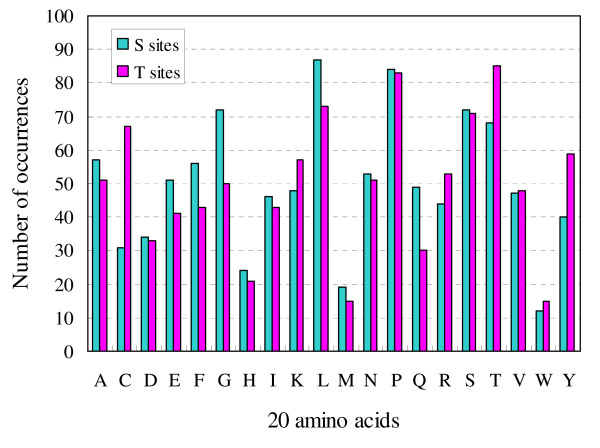
**The occurrences of each amino acid in top 500 *k*-spaced amino acid pairs**. The top 500 *k*-spaced amino acid pairs were resulted from the CC-based feature selection and ranked by considering all the cross-validation tests.

To compare amino acid usage in the top ranked *k*-spaced amino acid pairs between S and T sites, the histogram of occurrences of each amino acid in the top ranked 500 *k*-spaced pairs was plotted in Figure 1. This histogram clearly showed that amino acid usage in the selected 500 *k*-spaced amino acid pairs is similar between S and T sites, suggesting that it is reasonable to integrate the prediction of S and T sites into one predictor. To materialize this concept, we merged the datasets of S and T sites to construct a new predictor. In this new predictor (i.e. S+T predictor), we combined central S and T to create a new amino acid type. The same encoding scheme, prediction method and performance assessment were then carried out. The new predictor was also tested in datasets with two different ratios of O-glycosylation to non-glycosylation sites (i.e. 1:1 and 1:5) and the results were summarized in Table 4. Generally, the performance of this new predictor is slightly better than those predictors where S and T sites were independently predicted (*cf*. Tables 1, 2 and 4), suggesting that there are no significant differences in the molecular recognition of O-glycosylated S/T sites and the increase of the method's accuracy may be a result of expanded training datasets. On the other hand, only one predictor is required to predict O-glycosylated S/T sites in this new strategy, which may be more convenient for practical use.

**Table 4:** Performance of S+T predictor^a^

Datasets^b^	Encoding	*Sn *(%)	*Sp *(%)	*Ac *(%)	*MCC*
1:1	CKSAAP^c,d^	82.9 ± 1.3	83.4 ± 1.8	83.2 ± 1.6	0.667 ± 0.033
1:5	CKSAAP^c,d^	63.7 ± 1.7	95.1 ± 0.3	89.8 ± 0.4	0.617 ± 0.017

^a ^In this predictor, the datasets of S and T sites were combined to train a prediction model. Therefore, the O-glycosylated S/T sites can be predicted in one predictor. ^b^The predictor was trained and tested in datasets with two different ratios of O-glycosylation and non-glycosyaltion sites (i.e. 1:1 and 1:5). ^c^The corresponding measurement was represented as the average value ± standard deviation. ^d^No feature selection method was applied.

Further analysis on the top *k*-spaced amino acid pairs may strengthen our understanding on the characteristics of the sequence surrounding O-glycosylation sites. As shown in Table 3, P, S and T frequently occur in these important amino acid pairs, which are in line with the observation that P, S and T residues frequently appear in the vicinity of O-glycosylation sites [14]. As reported by Christlet and Veluraja [24], P at +3 and/or -1 positions strongly favors O-glycosylation sites, which is also correlated with our analysis that SXXP, TXXP, PS and PT are top ranked amino acid pairs (*cf*. Table 3). Moreover, the listed amino acid pairs also support the observation that the residues with small side chains are preferred to be located in O-glycosylation sites [14].

### Comparison of different encoding schemes

**Figure 2:**
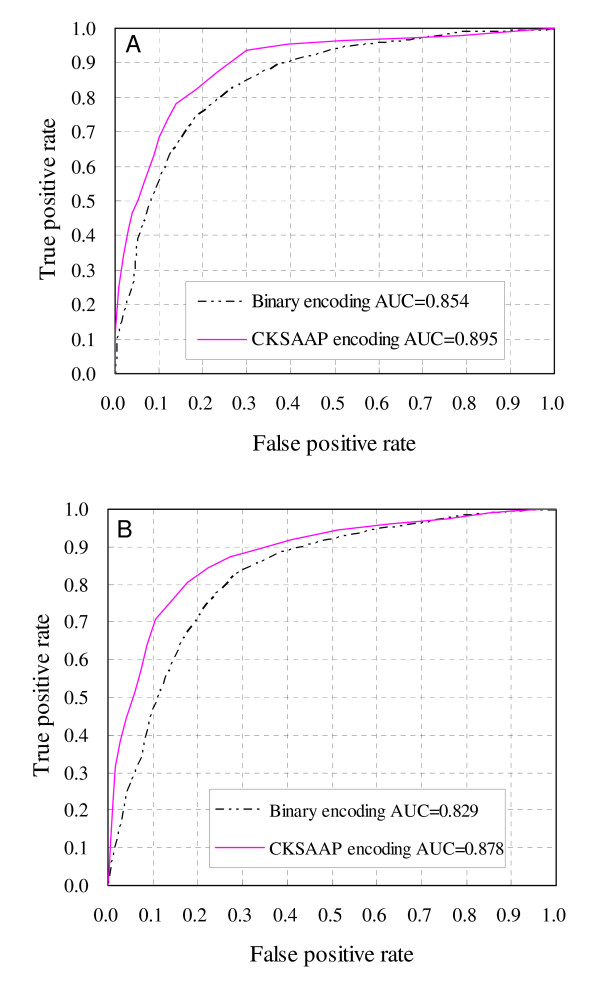
**ROC curves of O-glycosylation site prediction based on balanced datasets**. (A) Prediction of O-glycosylated S sites. (B) Prediction of O-glycosylated T sites. No feature selection was carried out for the CKSAAP encoding.

**Figure 3:**
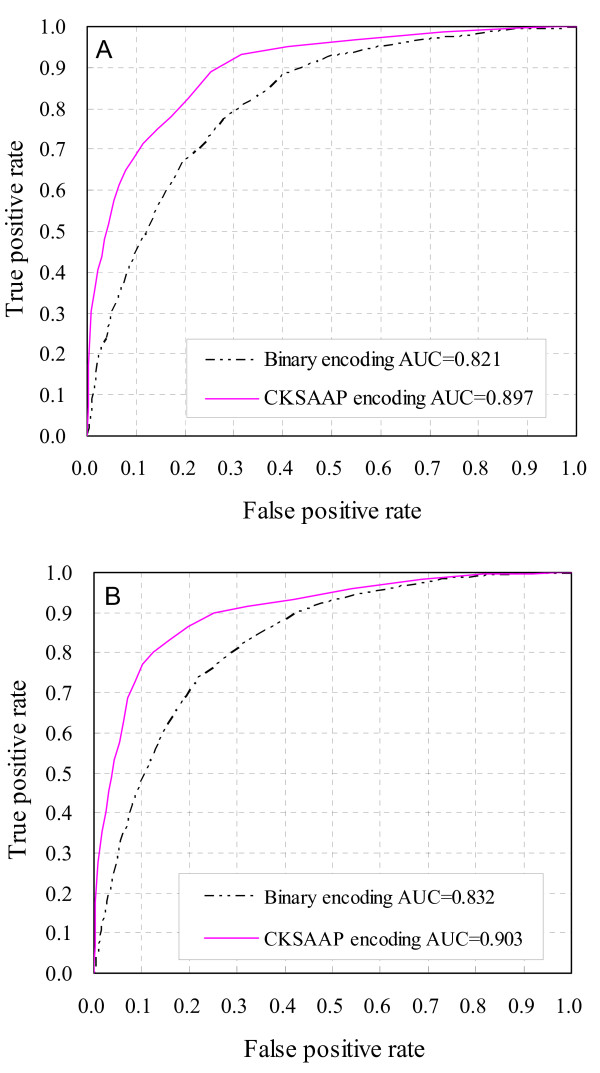
**ROC curves of O-glycosylation site prediction based on 1:5 datasets**. (A) Prediction of O-glycosylated S sites. (B) Prediction of O-glycosylated T sites. No feature selection was carried out for the CKSAAP encoding.

When the predictors were trained and tested in balanced datasets, the CKSAAP encoding revealed about 5.0% higher accuracy than the binary encoding (*cf*. Table 1). While in 1:5 datasets, the CKSAAP encoding revealed an even more significant improvement than the binary encoding. In contrast to the binary encoding, the CKSAAP encoding showed an increased *MCC *value of 0.211 and 0.192 in predicting S and T sites, respectively (*cf*. Table 2). The comparison was further illustrated in the Receiver Operating Characteristic (ROC) curves (Figures 2 and 3). The ROC curves of the prediction of O-glycosylated S and T sites based on balanced datasets were shown in Figures 2A and 2B, while the ROC curves based on 1:5 datasets were illustrated in Figures 3A and 3B. Generally the highest and leftmost ROC curve in the plot represents the best classification method. As shown in Figures 2 and 3, the results based on CKSAAP encoding scheme are much better than those based on the classical binary encoding, which can be further quantified by the corresponding areas under ROC curves (AUC). Either in predicting S or T sites, the AUC resulted from CKSAAP encoding based on balanced datasets is about 0.04–0.05 higher than that of binary encoding. While in 1:5 datasets, the CKSAAP encoding yielded about a 0.07 increase of AUC than the binary encoding. When benchmarked on balanced datasets without the requirement of a <40% sequence identity between positive and negative sites, the CKSAAP encoding also revealed about 5.0% higher accuracy than the binary encoding (*cf*. Table 1). Overall, the above results clearly showed that the CKSAAP encoding has a significant advantage over the binary encoding in predicting O-glycosylation sites.

In this study, a sequence identity of 40% was initially used to remove the data redundancy within the datasets. Since the above prediction results clearly showed that the composition of amino acid pairs surrounding O-glycosylation sites is more important than the sequence of the flanking segments, it would be interesting to check the results when the amino acid composition is used for exclusion of similar sites. To perform such a computational experiment, a 0.95 correlation coefficient was used as the cut-off to filter our datasets (i.e. in case the correlation coefficient of any two sequence segments' amino-acid composition is larger than 0.95, only one segment is kept), and the other procedures were the same as the sequence identity based filtration. After the filtration, the final positive dataset including 85 glycosylated S and 164 glycosylated T sites and the final negative dataset containing 938 non-glycosylated S and 1494 non-glycosylated T sites were obtained. The same prediction method and performance assessment were carried out on the new datasets. The results showed that the performance of CKSAAP encoding is better than the binary encoding (+4.6% and +4.1% accuracy improvement in predicting S and T sites) (*cf*. Table 5). It is confirmed that the CKSAAP encoding is more powerful than the binary encoding in predicting O-glycosylation sites.

**Table 5:** Prediction performance based on the datasets filtered by amino acid composition^a,b^

Site	Encoding scheme	*Sn *(%)	*Sp *(%)	*Ac *(%)	*MCC*
S	Binary^c^	73.9 ± 3.8	83.1 ± 5.9	78.5 ± 3.2	0.590 ± 0.068
	CKSAAP^c,d^	79.3 ± 2.0	86.8 ± 2.0	83.1 ± 1.8	0.677 ± 0.032

T	Binary^c^	77.7 ± 2.7	83.1 ± 3.0	80.4 ± 2.5	0.612 ± 0.052
	CKSAAP^c,d^	81.1 ± 1.8	88.0 ± 1.1	84.5 ± 1.1	0.699 ± 0.023

^a ^The predictors were based on datasets with a 1:1 ratio of O-glycosylation to non-glycosylation sites. ^b^The cut-off value of correlation coefficient between any two sequence segments' amino acid composition was set as 0.95. ^c^The corresponding measurement was represented as the average value ± standard deviation. ^d^No feature selection method was applied.

Why the CKSAAP encoding is better than the binary encoding in predicting O-glycosylation sites? The question may be answered from the following aspects. The binary encoding clearly characterizes amino acids in different positions surrounding a potential glycosylation site, but it is weak in reflecting the coupling effect of amino acid pairs at different positions. On the other hand, the CKSAAP pays attention on the correlation of amino acid pairs at different positions, but position specific amino acid information can not be inferred from the CKSAAP alone. It has been well known that there was no consensus motif identified for the neighbouring residues around O-glycosylation sites, but some frequently occurred amino acids were observed. Therefore, the CKSAAP encoding is particularly suitable for the prediction of O-glycosylation. Additionally, a similar conformation may be generally required by O-glycosylation sites. For example, it has been well established that O-glycosylation sites are preferred in coil or turn regions either situated near the termini of proteins, or in linker regions between domains [8]. The CKSAAP encoding can elegantly reflect short-range interactions of amino acids and it is very informative in predicting the local conformation of a sequence fragment [25]. That is probably another reason why the CKSAAP encoding can surpass the binary encoding in predicting O-glycosylation sites.

### Comparison of CKSAAP_OGlySite with other predictors

The proposed CKSAAP_OGlySite method was benchmarked against NetOGlyc 3.1 [8], one of the best O-glycosylation site predictors. The benchmark was based on 1:5 datasets, almost the same ratio as used in NetOGlyc 3.1. To perform a comparison, all the testing examples in 1:5 datasets were submitted to the NetOGlyc 3.1 server [31] and the average prediction accuracy was also calculated.

The performance of our method is significantly better than that of NetOGlyc 3.1 by showing about 0.102 and 0.059 higher *MCC *value in predicting O-glycosylation S and T sites, respectively (*cf*. Table 2). It should be pointed out that some testing examples were possibly already selected in training NetOGlyc 3.1. Since the developers of NetOGlyc 3.1 did not distribute their training data set publicly, we were not able to exclude these examples from the analysis. In case the comparison is based on a completely independent dataset, the increased accuracy resulted from our method may be more significant. As reported in the paper of NetOGlyc 3.1 [8], different encoding schemes were jointly employed, including the binary encoding, predicted structural information and evolutionary information inferred from PSI-BLAST search. Noted that the structural properties used in NetOGlyc 3.1 were predicted from the sequence information with the assistance of other programs, the major input of NetOGlyc 3.1 is the sequence context of O-glycosylation sites and the corresponding evolutionary information. The sequence conservation is not highly required for O-glycosylation sites, the power of evolutionary information is limited [6]. Using the current training and testing datasets, we also benchmarked the evolutionary information based encoding, and the result is only slightly better than that of binary encoding (data not shown). Therefore, it is reasonable that our CKSAAP_OGlySite is able to provide better performance than NetOGlyc 3.1.

The proposed CKSAAP_OGlySite method was also benchmarked against OGlyC method, a SVM-based O-glycosylation site predictor [18]. When trained and tested in balanced datasets, OGlyC based on the binary encoding scheme reached an accuracy of 85.0% [18]. Using the similar strategy for selecting the positive and negative sites, the same ratio of positive and negative sites (1:1), the same encoding scheme, window size (*i.e*. 2*n*+1 = 41) and machine learning method (*i.e*. SVM), the prediction accuracy of our method based on the binary encoding is much less impressive (about 78.0% accuracy in predicting S sites and 76.6% accuracy in predicting T sites) (*cf*. Table 1). The selection of training dataset in our method is based on a newer version of the Swiss-Prot database. The accuracy difference may be resulted from the different selection of datasets, especially the selection of negative sites. Using the same datasets and the same cross-validation, it has been clearly proved that the CKSAAP encoding based SVM model has a much higher accuracy than that of the binary encoding. Given the same datasets, the performance of our CKSAAP_OGlySite should be better than that of OGlyC.

With more and more O-glycosylation sites experimentally verified, we hope some standard training and testing datasets will be available in the near future. Thus, different prediction methods can be reliably benchmarked. Meanwhile, some well-established strategies in assessing different protein structure prediction methods (e.g. Live-Bench [32] and EVA [33]) should also be considered in evaluating different O-glycosylation site predictors.

## Conclusion

A competitive mucin-type O-glycosylation site predictor named as CKSAAP_OGlySite has been developed in the present study. The proposed CKSAAP_OGlySite demonstrated higher prediction accuracy than some other existing predictors, although the overall accuracy is still not satisfactory and there is possibility to develop more accurate predictors in the foreseeable future. With the ability of reflecting the characteristics of the sequence surrounding the O-glycosylation sites, the CKSAAP encoding has been proved to be particularly suitable for the prediction of O-glycosylation sites. By using other state-of-the-art machine learning methods as well as combining other encoding schemes, it is expected the CKSAAP encoding can play an important role in developing new O-glycosylation site predicting systems.

To facilitate the biological community, a web-server of CKSAAP_OGlySite was constructed, which can be used for proteome-wide O-glycosylation site prediction. Since the training dataset used in the current method is merely based on a limited number of experimentally verified O-glycosylated proteins, it should be pointed out that the performance for proteome-wide prediction may be less impressive in comparison to the accuracy reported in this paper. On the other hand, if we have the prior knowledge that query proteins are known to be O-glycosylated, the prediction of such proteins may result in an expected accuracy close to the value reported in this paper.

## Methods

### Datasets

The experimentally validated mucin-type O-glycosylation sites from mammalian proteins were extracted from the Swiss-Prot database (Release 52.4)[30], which contains 103 proteins covering 125 S and 242 T sites, and were compiled into two positive datasets (Pos_S and Pos_T). Each site within the datasets is represented by a sequence fragment of 41 amino acids, where S or T is in the central position. For the sites located in N- or C-terminus, the number of upstream or downstream residues may be less than 20. To ensure a sequence fragment with a unified length, we assigned a non-existing amino acid O to fill in the corresponding positions. Thus, 21 different amino acids are considered in the present study to reflect the sequence context of a glycosylation site, which are ordered as ACDEFGHIKLMNPQRSTVWYO. To remove redundant fragments within the datasets, the initial datasets (Pos_S and Pos_T) were further filtered by a 40% sequence identity cut-off. Since each site is represented by a sequence fragment with fixed length, the sequence identity is simply based on the match between two fragments (*i.e*. no-gap alignment). Considering the middle residue in each fragment is always the same (S/T), the central position is excluded when calculating the sequence identity, meaning that only sixteen residues are maximally allowed to be identically matched in the alignment. The similar filtration method was previously used for the preparation of training datasets in the prediction of phosphorylation sites [34,35]. Thus, our final positive datasets included 116 S and 212 T respectively [see Additional file 1 and Additional file 2]. It should be emphasized that the annotation of Swiss-Prot was regarded as a golden standard for selecting positive O-glycosylation sites, and the original publications for these O-glycosylation sites were not checked. Due to the potential annotation errors, the quality of the compiled O-glycosylation dataset was inevitably limited by the knowledge of Swiss-Prot database.

All S and T residues in these 103 protein sequences with no annotation related to O-glycosylation site were selected as negative sites. In the present study, 1506 non-glycosylated S residues and 2529 non-glycosylated T residues were initially selected, and were further compiled into two negative datasets (Neg_S and Neg_T). Likewise, we also filtered the negative data sets using a 40% sequence identity to avoid the redundancy. Furthermore, the negative site sharing over 40% identity with any of the positive sites was also discarded. Finally, we got 1153 non-glycosylated S and 1702 non-glycosylated T residues [see Additional file 3 and Additional file 4].

### Feature construction

A new feature construction, the composition of *k*-spaced amino acid pairs (CKSAAP) based encoding, was employed. The detailed procedures are described as follows. Generally, a sequence fragment of 2*n*+1 amino acids (*i.e*. the window size is equal to 2*n*+1, and the maximal window size is 41 as defined in the section of Datasets) is used to define a glycosylation site. For *k*-spaced amino acid pairs (*i.e*. pairs that are separated by *k *other amino acids) within this sequence fragment, there are 441 possible types (AA, AC, AD, ..., OO). Then, a feature vector of that size is used to represent the composition of these pairs, which can be described as

(1) (*c*_*AA *_*c*_*AC *_*c*_*AD *_... *c*_*OO*_)_441_

The value of each feature denotes the composition of the corresponding amino acid pair in the fragment. For instance, if an AD pair occurs *m *times in this fragment, the corresponding value in the vector (*i.e.c*_*AD*_) is equal to *m*. The amino acid pairs for *k *= 0, 1, ..., *k*_*max *_are jointly considered in this study, so the total dimension of the proposed feature vector is 441 × (*k*_*max*_+1).

To benchmark the proposed CKSAAP encoding, the prediction based on the binary encoding was also carried out. In this encoding scheme, each amino acid is represented by a 21-dimensional binary vector, e.g. A (100000000000000000000), C (010000000000000000000), ..., O (000000000000000000001), etc. For a query O-glycosylation site represented by a fragment of 2*n*+1 residues, the central residue is always S/T, which is not necessary to be taken into account. Therefore, the total dimension of the proposed binary feature vector is 21 × 2*n*.

### Feature selection

Due to the high dimensionality as well as the sparse nature of the CKSAAP encoding, the dimensionality reduction seems to be required. CC- and IE-based dimensionality reduction methods, previously reported by Chen *et al*. [25], were employed in this work.

#### CC-based feature selection

For each variable from the CKSAAP based feature vector (*X*) and the known predicted variable (*Y*), the correlation coefficient *cor*(*X*,*Y*) is computed. The value of *cor*(*X*,*Y*) is in the range from -1 to 1. Higher value of | *cor*(*X*,*Y*)| means the corresponding variable *X *is more significantly correlated with *Y*. To reduce the dimensionality, therefore only those variables with higher | *cor*(*X*,*Y*)| were kept.

#### IE-based feature selection

For any variable *X *from the CKSAAP encoding, its information entropy is defined as

(2) $$I(X)=-\sum_{i}P(x_{i})\log_{2}(P(x_{i}))$$

Where {*x*_*i*_} represents a set of values occurred in *X*, and *P*(*x*_*i*_) denotes the prior probability of *x*_*i*_. The conditional entropy of *X *under the condition of *Y *is defined as

(3) $$I(X|Y)=-\sum_{j}P(y_{j})\sum_{i}P(x_{i}|y_{j})\log_{2}(P(x_{i}|y_{j}))$$

Where *P*(*x*_*i*_|*y*_*j*_) is the posterior probability of *x*_*i *_given the value *y*_*j *_of *Y*. Then, information gain *IG*(*X*|*Y*) is given by

(4) *IG*(*X*|*Y*) = *I*(*X*) - *I*(*X*|*Y*)

The information gain *IG*(*X*|*Y*) indicates the additionally increased information about *X *provided by *Y *[25]. For any two features (*X1 *and *X2*) from the CKSAAP encoding, if *IG*(*X*1|*Y*) > *IG*(*X*2|*Y*), the feature *Y *is regarded as more correlated with *X1 *than *X2*. To reduce the dimensionality, therefore the features with higher *IG *are selected.

### Support Vector Machine (SVM)

The SVM is a machine-learning algorithm for two classes of classification with the goal to find a rule that best maps each member of training set to the correct classification [36], which has been widely used in the field of protein bioinformatics [37-41]. In linearly separable cases, SVM constructs a hyperplane that separates two different groups of feature vectors in the training set with a maximum margin. The orientation of a test sample relative to the hyperplane gives the predicted score, and hence the predicted class can be derived. The implementation of SVM algorithm used in this work was SVM-Light [42]. The applied kernel functions were the linear function, polynomial function, and radial basis function (RBF). The selection of the kernel function parameters is important for SVM training and testing, because it implicitly determines the structure of the high dimensional feature space when constructing the optimal hyperplane [43]. In the current study, several parameters need to be determined in advance to optimize SVM training, such as the regularization parameter *C*, which controls the trade-off between training error and margin, the width parameter *γ *in the RBF kernel $K({\vec{X}}_{i},{\vec{X}}_{j})=\exp(-\gamma {\left\|{\vec{X}}_{i}-{\vec{X}}_{j}\right\|}^{2})$, and the degree *d *in the polynomial kernel $K({\vec{X}}_{i},{\vec{X}}_{j})={\left({\vec{X}}_{i}•{\vec{X}}_{j}+\text{1}\right)}^{d}$. Other than changing the kernel functions and the necessary regulation of the kernel function parameters, the algorithm was run with the default settings in a Linux Platform.

### Performance assessment

In this study, two subsets (Neg_S_Sub and Neg_T_Sub) were randomly constructed from Neg_S and Neg_T to have the same size as Pos_S and Pos_T, respectively. Each set of Pos_S and Pos_T with the corresponding negative sets of Neg_S_Sub and Neg_T_Sub was used to construct predictors for S and T sites. Then, a 10-fold cross-validation was performed. To check the difference of predictive accuracy caused by the different choices of negative data sets, the above 10-fold cross-validation was repeated 5 times by randomly changing the negative datasets (*i.e*. Neg_S_Sub and Neg_T_Sub). Finally, the overall performance was averaged over these 5 times of 10-fold cross-validation tests. Thus, the current cross-validation generally reflected the overall performance of the proposed method over the selected data sets. The same training and testing procedures were used in assessing the binary encoding based predictor.

Similar to the above procedures, datasets with 1:5 ratio of positive to negative sites were also used to train the proposed predictors. Then, 10-fold cross-validation tests were carried out. The negative dataset was also randomly changed for five times and the average prediction accuracy was obtained.

Four measurements, *i.e. Ac*, *Sn*, *Sp *and *MCC*, were used to evaluate the prediction performance with definitions as below:

(5) $$Ac=\frac{TP+TN}{TP+FP+TN+FN},$$

(6) $$Sn=\frac{TP}{TP+FN},$$

(7) $$Sp=\frac{TN}{TN+FP},$$

and

(8) $$MCC=\frac{\left(TP\times TN)-(FN\times FP\right)}{\sqrt{\left(TP+FN)\times (TN+FP)\times (TP+FP)\times (TN+FN\right)}}.$$

TP, FP, FN and TN denote true positives, false positives, false negatives and true negatives.

The prediction accuracy was also measured by using the ROC analysis [44,45]. For a prediction method, the curve of ROC plots true positive rate (*i.e*. *Sn*) as a function of false positive rate (*i.e*. 1-*Sp*) for all possible thresholds. The AUC was also calculated to provide a comprehensive understanding for the proposed prediction method. Generally, the closer the AUC value is to 1, the better the prediction method is.

## Availability and requirements

**Project Name**: CKSAAP_OGlySite predictor

**Project home page**: 

**Operating system**: Online service is web based; local version of the software [see Additional file 5] should be run in Linux platform.

**Programming language**: Perl.

**Other requirements**: None.

**License**: Free.

**Any restrictions to use by non-academics**: None.

## Authors' contributions

YZC collected data, wrote codes and developed the web server. YRT and ZYS participated in the research design, method assessment and preparation of the manuscript. ZZ directed the research and wrote the manuscript. All authors read and approved the final manuscript.

## Supplementary Material

Additional file 1 O-glycosylated S sites. This file contains the positive dataset of O-glycosylated S sites used in training and testing the proposed CKSAAP_OGlySite predictor. Click here for file

Additional file 2 O-glycosylated T sites. This file contains the positive dataset of O-glycosylated T sites used in training and testing the proposed CKSAAP_OGlySite predictor. Click here for file

Additional file 3 Non-glycosylated S sites. This file contains the negative dataset of S sites used in training and testing the proposed CKSAAP_OGlySite predictor. Click here for file

Additional file 4 Non-glycosylated T sites. This file contains the negative dataset of T sites used in training and testing the proposed CKSAAP_OGlySite predictor. Click here for file

Additional file 5 The source code of CKSAAP_OGlySite. The data provided contain the source code of CKSAAP_OGlySite, in which a file (readme.txt) addressing how to use CKSAAP_OGlySite is included. Click here for file
